# Development of a Rapid and Non-Destructive Method for the Detection of Water Addition in Octopus Species (*Octopus vulgaris* and *Eledone cirrhosa*) Using Time Domain Reflectometry (TDR)

**DOI:** 10.3390/foods12071461

**Published:** 2023-03-29

**Authors:** Bárbara Teixeira, Helena Vieira, Sandra Martins, Rogério Mendes

**Affiliations:** 1 Portuguese Institute for the Sea and Atmosphere, Department for the Sea and Marine Resources, Avenida Doutor Alfredo Magalhães Ramalho, 6, 1495-165 Algés, Portugal; helenavieira27@gmail.com (H.V.); sandra.regalado@ipma.pt (S.M.); rogerio@ipma.pt (R.M.); 2 Interdisciplinary Center of Marine and Environmental Research (CIIMAR), University of Porto, Rua das Bragas 289, 4050-123 Porto, Portugal

**Keywords:** rapid methods, dielectric properties, water addition, fraud, cephalopods

## Abstract

Consumer expectations regarding the quality of octopus are often frustrated and dissatisfaction is frequent, namely due to the excessive reduction in weight after cooking. Therefore, a rapid and non-destructive method based in time domain reflectometry (TDR) was developed for the control of water added to octopus (*Octopus vulgaris* and *Eledone cirrhosa*). *O. vulgaris* had significant higher values of moisture content, moisture/protein ratio, and cooking loss than *E. cirrhosa*. Immersion in freshwater increased the weight of *O. vulgaris* in ca. 32% after 32 h, and of *E. cirrhosa* in ca. 21% after 36 h, and cooking losses increased about 13.9% and 26.1%, respectively. The results reveal how consumers can be misled by abusive water addition. Changes in electrical conductivity and TDR curves were linked with the increasing incorporation of water and dilution effect of salts from octopus muscle. TDR technology and linear discriminant analysis were combined to detect added water in octopus. The classification model developed was cross-validated and 98.6% of samples were correctly classified. The method can be used to proof the authenticity of octopus (*O. vulgaris* and *E. cirrhosa*) or to detect fraudulent practices regarding added water.

## 1. Introduction

Due to its premium price, seafood is susceptible to mislabeling [1,2,3], and fraudulent addition of water to fishery products is a practice more and more commonly encountered worldwide in the seafood industry [1,4,5,6]. Labelling rules ratified in the European Union in terms of the obligatory Declaration of the Quantity of Ingredients in food products allow consumers to have access to a wide range of data about the ingredients and composition of foodstuffs, and aid citizens to make wiser decisions when buying food products. Regulation (EU) No 1169/2001 [7] states that “…the name of the food shall include an indication of the presence of added water if the added water makes up more than 5% of the weight of the finished product… in the case of fishery products and prepared fishery products which have the appearance of a cut, joint, slice, portion, filet or of a whole fishery product”.

Cephalopods of the genus Octopus spp. are among the most widely distributed and commercially important octopus species harvested worldwide [8]. The European market is one of the most important in the world for cephalopods, and particularly in southern European countries, the common octopus (*Octopus vulgaris*) is one of the most important fishery resources in terms of economic value. Fresh and frozen octopus attain high prices throughout the whole of the distribution chain due to diet traditions, thus sustaining artisanal as well as industrial fisheries [9]. Curled octopus (*Eledone cirrhosa*), a lower-priced species captured as bycatch in trawl fisheries, is also relevant in the south European markets and is a common substitute of *O. vulgaris*. Although the demand for products is high, consumer expectations regarding the quality of the octopus purchased are not always met and dissatisfaction is frequent, namely due to the excessive reduction in weight/volume after cooking, a common result of which is the reduction of cooked octopus to less than half the acquired weight [5]. In recent years, media reports of food fraud and in particular seafood counterfeiting have increased, and a variety of incidents have been reported to defraud the public, restaurants, retailers and other seafood businesses [10,11]. Induced by high prices and consumers’ frustration about the low yield of these products after cooking, suspicions regarding excessive water content of octopus, which could be regarded as adulteration involving undeclared water addition and economic fraud for the buyers, have been published [5,12,13]. These procedures, not supported by any technological need, are generating on the one hand an unfair competition between producers, to the extent that the prices posted by the fraudsters are often more attractive; on the other hand, they deceive consumers, who buy water at the price of seafood.

Current official control methods for water addition determination in seafood rely on the strong physiological correlation existent between protein and moisture levels [14,15]. Knowledge of the baseline levels of proximate composition of unprocessed products and the use of lengthy and destructive analytical methods involving determination of moisture and protein support the present official control of excessive water addition in commercial products [1,16]. However, for labelling control and quick determination of fraudulent practices in the industry, or in commercial products available fresh in markets, the ideal method for moisture analysis should be fast, appropriate to a varied array of food products and ingredients, safely doable by a nontechnical person after minimal training and use inexpensive and readily available equipment, while having good precision and accuracy [17,18]. Direct (destructive) and indirect (non-destructive) methodologies for quantification of the moisture present in food products have been extensively reviewed by Park and Bell [19]. Within these, non-destructive methods are gaining wide attention due to their advantages such as speed, on-site usability and high accuracy.

A new method based on the use of microwave dielectric spectroscopy has been proposed to determine water content in foods, as an alternative to conventional methods [20]. As water is the major constituent of food and a molecule with an electric dipole moment, the interaction between water molecules and oscillating electric fields in the scale of microwaves disturbs the dynamic vibration modes (symmetric stretching, asymmetric stretching and bending) of these molecules and impacts the dielectric spectrum of the microwave [21]. Changes in the quantity of water or the nature of its binding to food are thus foreseen to alter the dielectric spectrum in the microwave region at a specified range of frequency, and its use has been proposed in several food products for different measurements, including moisture [20,22,23]. Time domain reflectometry (TDR) is therefore a methodology based in the principles of the microwave dielectric spectroscopy in which the dielectric properties of the material are probed in a wide range of microwave frequencies (100 MHz–10 GHz), the electric current (voltage) returned by the sample is measured in function of time, and multivariate analysis applied to the corresponding spectra [24]. This method has several advantages: it is non-destructive, easy, quick, effective, reliable and practical and also offers the possibility of application in smart devices and automation for on/in-line determinations of quality [25].

Mendes et al. [16] proposed a method for the detection of water addition to *O. vulgaris* based in the measurement of the dielectric properties in the microwave region as a function of frequency. Likewise, and targeting the analysis of water illegally added to octopus, Teixeira et al. [26] further developed a fast and non-destructive method involving time domain reflectometry analysis (TDR), which allows the detection and quantification of the added water specifically in *O. vulgaris*. According to the authors, the correlation found between the TDR data and the moisture content evidences the potential of this nondestructive methodology as a tool for the public quality control regulators and industry stakeholders to assess the quality of octopus and its conformity with regulations. Lee et al. [12] also reported that the analysis of the dielectric properties of *Octopus minor*, one of the commercially important marine species in East Asian countries, using the open-ended coaxial probe method and direct contact, allowed the differentiation of artificially water-injected frozen octopus from normally frozen octopus.

This study aims to develop a fast and non-destructive method of time domain reflectometry analysis (TDR) combined with multivariate statistics for the control/detection of water addition in two octopus species, common octopus *O. vulgaris* and curled octopus *E. cirrhosa*. Octopus samples immersed in freshwater for different periods were analyzed with TDR, and the data used to develop and validate a classification model. It is intended that the development of this analysis method will simplify the control of the abusive practice of adding water to *O. vulgaris*, *E. cirrhosa*, and also in mixed/unknown octopus products, including by non-trained personnel. Additionally, the study aims to characterize the baseline levels of moisture, protein, electrical conductivity, and cooking losses of octopus for control quality purposes and to compare the kinetics of water uptake in these two octopus species.

## 2. Materials and Methods

### 2.1. Raw Materials, Processing, and Sampling

This study was carried out with common octopus *Octopus vulgaris* and curled octopus *Eledone cirrhosa* captured in Peniche and Aveiro (Portugal), respectively, during the period 2019–2021. Six trials (*n* = 77) were carried out with *O. vulgaris*, and the number of specimens varied between 12 and 14 in each trial. In the case of *E. cirrhosa*, three trials (*n* = 44) were carried out, using between 12 and 20 specimens in each trial. The weight of individuals was 1032 ± 377 g and 297 ± 91 g for *O. vulgaris* and *E. cirrhosa*, respectively.

Water addition trials followed the previous experience of Mendes et al. [27]. To suppress individual variability during water addition studies, each octopus was divided into four equal sections, and each section comprising two arms and the corresponding portion of the head was tagged, numbered, and processed. One of these sections was always used as a control, while the corresponding remaining three sections were subjected to three different treatments. *O. vulgaris* samples were immersed in freshwater (sample:water 1:2 *w*:*v*) for different periods of time (between 0.5 h and 36 h) in a refrigerated chamber (3 ± 1 °C). The various treatments were intended to give a wide range of water uptake values in octopus samples. TDR analysis was performed using RFQ-Scan^®^ equipment (Bremen, Germany) (see Section 2.4) in all octopus samples (control and water-added), and then samples were vacuum-packed and frozen (−20 °C) until further analysis (e.g., moisture and protein).

**Figure 1:**
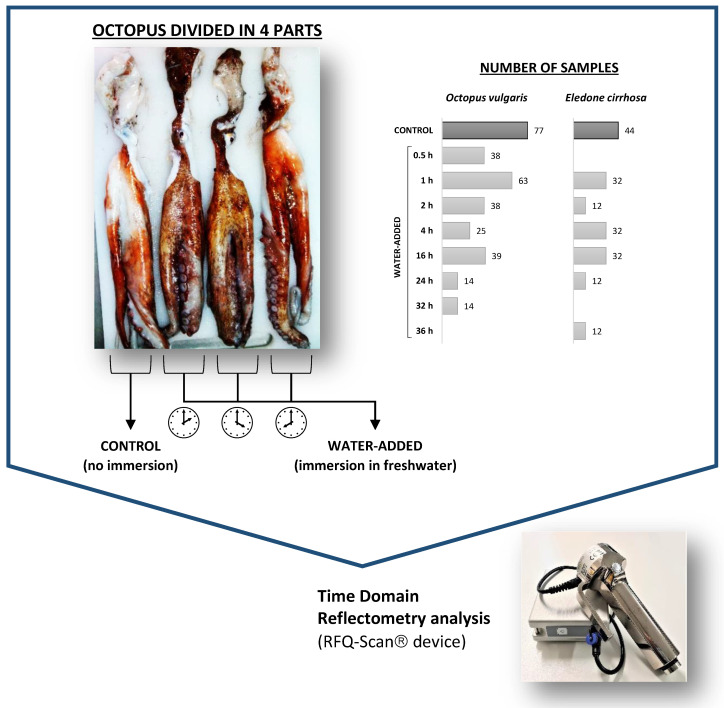
Schematic representation of water addition trials with *Octopus vulgaris* and *Eledone cirrhosa*. Each octopus was divided into four parts, one part being used as the control, and the three remaining parts being immersed in freshwater for different periods of time (between 0.5 h and 36 h). Time domain reflectometry analysis was carried out in control and water-added octopus samples with an RFQ-Scan^®^ device.

A schematic representation of the water addition trials performed with both octopus species is shown in Figure 1. The number of samples used for each treatment is also specified in Figure 1.

### 2.2. Weight Changes, Cooking Loss, Moisture and Protein Contents

Weight changes were calculated as percentage weight differences of octopus samples before and after water addition trials [27]. Cooking losses were calculated as the percentage in weight differences between the raw and cooked octopus based on the raw weight, according to Mendes et al. [27]. Analysis of cooking loss was carried out in samples vacuum packed in 140 mm thickness polyamide and polyethylene film bags (Vaessen-Schoemaker, Ovar, Portugal) and cooked in a pre-heated steam oven Rational Combi-Master model CM6 (Landsberg, Germany) for 60 min at 100 °C. After thermal processing, the samples were first left until reach the room temperature (25 °C) and then weighted.

Moisture content was determined by standard gravimetric analysis [28]. Crude protein content was determined by the Dumas combustion method according to Saint-Denis and Goupy [29] in a LECO FP-528 protein/nitrogen analyzer (LECO Corp., St Joseph, MI, USA), calibrated with ethylene diamine tetraacetic acid. All determinations were carried out in duplicate.

### 2.3. Electrical Conductivity

Owing to the non-availability of dedicated surface conductivity probes specifically designed for non-destructive octopus analysis, alternative methods adapted to be as close as possible to the objective intended were used. Electrical conductivity was measured in 10 g of octopus muscle homogenized with 10 mL of Merck-Millipore Milli-Q water (Darmstadt, Germany) in a Polytron 5000 blender (40 s, 15,000 rpm). An Orion 162 conductivity meter equipped with an Orion 018010 two-electrode electrical conductivity cell (Orion Research Inc., Boston, MA, USA) was used for electrical conductivity determinations. All measurements were made in duplicate.

### 2.4. Time Domain Reflectometry—RFQ-Scan^®^ Analysis

Analysis of TDR data was carried out following the method described by Teixeira et al. [26]. RFQ-Scan^®^ (Radio Frequency Quality Scan) equipment produced and patented by Sequid GmbH (Bremen, Germany) was used. This handheld detector produces an electrical signal with a step-like voltage increase of around 100 ps and total duration of 2.56 ns, which is conducted to the samples via an open-ended probe. Statistical methods using multivariate analysis are then used to evaluate the reflected signals in the domain of time and in a relatively wide bandwidth of around 5 GHz. More details of the time domain spectrometer were reported by Schimmer and Knöchel [30]. The temperature of the samples was kept low and almost constant during preparation, and prior to measurements (3 ± 1 °C), for which its effect was assumed to be minor. Eight measurements were made in different parts of the octopus skin.

### 2.5. Statistical Analysis

A general linear model (one-way analysis of variance, ANOVA) was used to determine significant differences in moisture, protein, M/P ratio, electrical conductivity, and cooking losses among different immersion time treatments. Multiple comparisons were carried out by Tukey’s honest significant difference test to identify the differences. In addition, differences between the two octopus species were tested using a *t*-test for independent samples. Statistical analyses were tested at a 0.05 significance level using the software STATISTICA Version 10 (StatSoft, Inc., Tulsa, OK, USA).

TDR data acquired with the RFQ-Scan^®^ device were pre-processed using MATLAB Version 7.6 (The Math-Works, Inc., Natick, MA, USA). Outliers were removed from the set of eight scans per sample, the measurements were averaged, and then a principal components analysis (PCA) was performed with the TDR data, as described by Schimmer et al. [31].

Linear discriminant analysis was performed using all principal components (a total of 24 principal components) obtained from TDR data between 0.6 and 1.5 ns. In a first approach, all trials/samples were used to create the classification model (model 1). In an alternative approach (model 2), two of the three trials were used for model training and one of the three trials was used for testing. A stratified five-fold cross-validation was performed, with the five groups comprising samples from all trials, both octopus species, and from all immersion time treatments to produce a correct distribution for each fold. The probability of a new sample belonging to one or another group was considered equal, without using any knowledge of the values for the variables in the model. Linear discriminant analysis was carried out with the software STATISTICA Version 10 (StatSoft, Inc., Tulsa, OK, USA).

## 3. Results and Discussion

### 3.1. Characterization of Control Octopus Samples

*O. vulgaris* and *E. cirrhosa* were characterized in terms of moisture and protein contents, moisture/protein ratio, and electrical conductivity. Control samples showed a moisture content of 82.2 ± 1.3 g/100 g with a variation range between 79.7 and 85.8 in the case of *O. vulgaris*, while *E. cirrhosa* had a significantly lower moisture content (78.9 ± 1.6 g/100 g), with a range between 76.1 and 82.4 g/100 g. It is important to note that an octopus sample with a low moisture content if adulterated with 20% of added water would still present a moisture content within the natural variation range. Additionally, considering that *E. cirrhosa* is a common substitute species of *O. vulgaris* and presents naturally lower moisture values than *O. vulgaris*, if adulterated with water, its moisture content would be within the natural moisture content range of *O. vulgaris*. Thus, it would probably not be considered nonconforming.

The moisture contents in *O. vulgaris* samples were comparable to results published previously [27,32,33], including if *O. vulgaris* was fed with different diets [34,35] (Table 1). In addition, although literature data on the proximate composition of *E. cirrhosa* are more limited, Spitz et al. [36] and Ruiz-Capillas et al. [37] reported moisture contents for *E. cirrhosa* within the range of values obtained in the present study (Table 1).

In contrast, higher moisture contents were observed for frozen octopus acquired in markets (Table 1), suggesting that commercial frozen octopus were adulterated with water. In particular, moisture contents of 85–90 g/100 g were frequent in commercial samples of frozen *O. vulgaris* [5,40,41], while values of 80–86 g/100 g were reported for *E. cirrhosa* [40,41].

For *O. vulgaris*, a data set with more than 300 samples from the Portuguese coast was used to propose a limit of moisture content for conformity assessment of commercial products (85.2 g/100 g), taking into account the highest values of the 95% confidence interval (84.4 g/100 g) and the 5% limit of added water [26]. In the case of *E. cirrhosa*, the 95% confidence interval obtained with the results of the current study (*n* = 44) was 75.7–82.1 g/100 g, and the limit of moisture content for conformity assessment proposed was 83.0 g/100 g.

The protein content in control samples was significantly lower in *O. vulgaris* (14.6 ± 1.2 g/100 g) than in *E. cirrhosa* (17.5 ± 1.3 g/100 g), with a variation range between 11.0 and 17.0 g/100 g and between 14.9 and 19.6 g/100 g, respectively. For comparison purposes, literature data on protein contents of different octopus species can also be found in Table 1. The previously published protein results of *O. vulgaris* varied between 12.0 and 19.8 g/100 g, e.g., [32,33], most falling within the range of values obtained in the present study. In the case of *E. cirrhosa*, protein results were also comparable to previously published data [36,37].

The moisture/protein (M/P) ratio was determined because it is an indicator of the presence of excess water in seafood products [43,44]. Baseline levels of M/P ratio were significantly higher in *O. vulgaris* (5.7 ± 0.6) than in *E. cirrhosa* (4.5 ± 0.4). Estimated M/P ratios based on average moisture and protein contents reported by several authors (Table 1) were comparable to results obtained in the current study (the variation range was 4.7–7.8 in *O. vulgaris* and 3.9–5.5 in *E. cirrhosa*).

Regarding electrical conductivity, *O. vulgaris* samples showed significantly lower electrical conductivity values than *E. cirrhosa* (*O. vulgaris*: 7.0 ± 1.4 mS.cm^−1^; *E. cirrhosa*: 7.6 ± 1.2 mS.cm^−1^), which is in line with the higher moisture content of *O. vulgaris* samples. Previously, higher electrical conductivity results (9.3 ± 0.7 mS.cm^−1^) were reported for fresh *O. vulgaris* [16]. The lower values obtained in the current study may be explained by the loss of salts during drip loss due to the freezing and thawing processes.

The results reported here for control samples of *O. vulgaris* and *E. cirrhosa* captured in Portugal can be used as a reference for the control of excessive water addition in commercial products.

### 3.2. Evaluation of Water Addition in Octopus Samples

**Figure 2:**
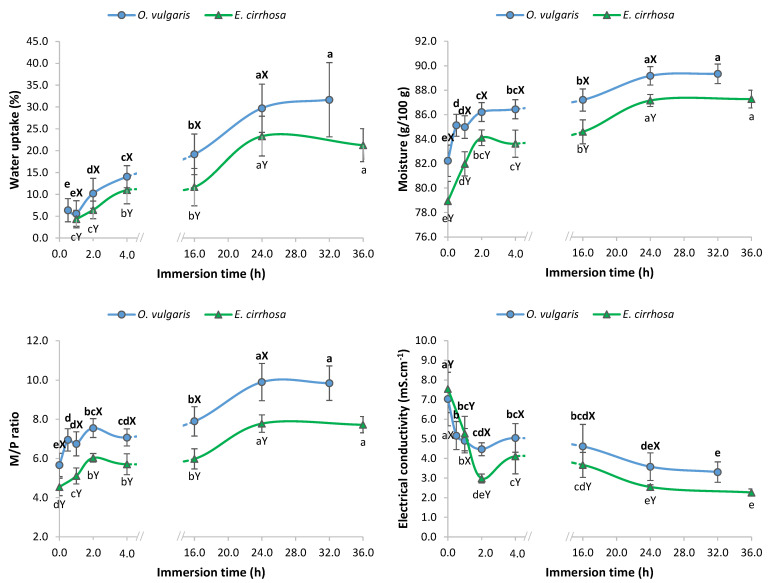
Effect of immersion time (0.5–36 h) in the water uptake, moisture content, moisture/protein (M/P) ratio, and electrical conductivity of *Octopus vulgaris* and *Eledone cirrhosa*. Different letters denote significant differences between species (X, Y) and different immersion time periods (a, b, c, …) for each species.

Immersion in freshwater for different periods of time was performed to study the water uptake of octopus and to obtain octopus samples with increasing moisture contents. According to legislation, if the added water corresponds to more than 5% of the weight of the finished product, then an indication of its presence must be included in the product label [7]. This limit is easily reached by immersing octopus in freshwater. Water uptake results showed that weight increases up to 5% occurred in both species immersed mainly in short treatments (0.5 h and 1 h) (Figure 2). Still, using such short processing times does not guarantee a weight increase lower or equal to 5%, as evidenced by the associated sample variability. The weight increase was significantly higher in *O. vulgaris* than in *E. cirrhosa* after 1 h of immersion (5.6 ± 2.9% and 4.3 ± 2.0%, respectively).

The weight increase obtained in the current study followed a similar trend to that reported previously for *O. vulgaris* immersed in freshwater up to 16 h [16,27]. In longer immersion time periods, the weight increase was 31.7 ± 8.5% in *O. vulgaris* after 32 h of immersion, while for *E. cirrhosa*, it was 21.3 ± 3.8% after 36 h of immersion. The maximum values observed were 51.7% and 29.6% for *O. vulgaris* and *E. cirrhosa*, respectively.

Weighing octopus before and after water-added treatments is an accurate way to determine if the 5% limit was attained. However, because added water has no technological role in octopus, industries have no interest in declaring that it has been added above that limit because it is carried out with fraudulent intentions. Thus, to control the conformity of octopus products regarding the established legislation, it was previously suggested to determine water uptake based on its relation with M/P ratio [27]. However, the results of the current study showed a higher dispersion of data, increasing in turn the error of estimated results (data not shown); therefore, this approach is not suitable, and alternative methods are needed.

Results regarding moisture content in octopus processed with freshwater for different periods of time are presented in Figure 2. The increase in immersion time caused an increase in moisture content, and moisture changes followed a similar trend in both octopus species, *O. vulgaris* and *E. cirrhosa*. Still, significant lower moisture contents were determined in *E. cirrhosa*. Water-added samples had moisture contents between 82.5 and 90.6% in the case of *O. vulgaris*, and between 80.0 and 88.5% in the case of *E. cirrhosa*. These ranges of values partially overlapped those obtained in control samples, which makes it difficult to distinguish between samples with and without added water based on moisture values.

After 16 h of immersion, moisture contents rose to 87.2 ± 0.9 g/100 g in *O. vulgaris* and to 84.6 ± 1.0 g/100 g in *E. cirrhosa*. Comparable values were reported previously for *O. vulgaris* after 16 h of immersion [27]. An extension of the immersion time significantly increased moisture content of octopus specimens. On average, the moisture content was 89.3 ± 0.8 g/100 g in *O. vulgaris* after 32 h of immersion, while for *E. cirrhosa*, it was 87.3 ± 0.7 g/100 g after 36 h of immersion. Similar values were also reported for several commercial frozen octopus (Table 1), thus indicating that industrials perform equivalent water addition treatments.

M/P ratio followed the same trend as moisture, with an increase in immersion time (Figure 2). *O. vulgaris* had higher M/P ratio values than *E. cirrhosa* regardless of immersion time. After 16 h, the M/P ratio values were 7.89 ± 0.75 in *O. vulgaris*, and 5.98 ± 0.51 in *E. cirrhosa*. Comparable M/P ratios (7.5 ± 0.6) were reported by Mendes et al. [27] for *O. vulgaris*. For extended immersion periods, M/P ratio values increased to 9.85 ± 0.88 in *O. vulgaris* after 32 h, and to 7.72 ± 0.42 in *E. cirrhosa* after 36 h. Similar M/P ratio values were reported for commercial frozen octopus (M/P ratio = 9.5 ± 1.9) available in markets from Portugal [5] (Table 1).

In general, the electrical conductivity decreased with the increase in immersion time (Figure 2). Although *E. cirrhosa* had higher electrical conductivity in control samples when compared with *O. vulgaris*, after 2 h of immersion, the values decreased to lower values than those of *O. vulgaris*. In a previous piece of research with fresh octopus, the electrical conductivity of *O. vulgaris* decreased to 3.6 ± 0.9 mS.cm^−1^, after 16 h of immersion in freshwater [16]. In comparison, in the current study, *O. vulgaris* had higher electrical conductivity (4.61 ± 1.12 mS.cm^−1^), while *E. cirrhosa* showed a similar value (3.67 ± 0.64 mS.cm^−1^) after 16 h. Electrical conductivity decreased even further with the increase in immersion time to values of 3.31 ± 0.52 mS.cm^−1^ in *O. vulgaris* after 32 h, and to 2.26 ± 0.18 mS.cm^−1^ in *E. cirrhosa* after 36 h. The decrease in electrical conductivity values is in line with the incorporation of water and dilution effect of ions in the octopus muscle.

### 3.3. Cooking Losses of Octopus Samples

**Figure 3:**
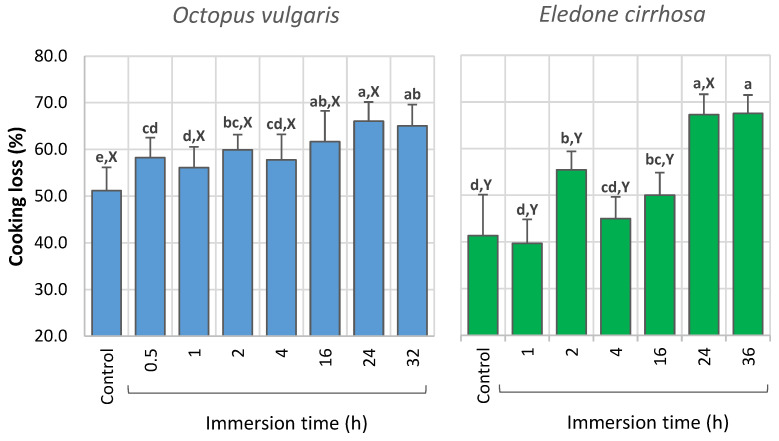
Cooking loss (%) of *Octopus vulgaris* and *Eledone cirrhosa* water-added samples immersed in freshwater for 0.5 h to 36 h. Different letters denote significant differences between species (X, Y) and different immersion time periods (a, b, c, …) for each species.

Consumers are very disappointed with cooking losses in commercial octopus. The results obtained in the current work showed that both octopus species naturally lose a substantial amount of water during cooking (Figure 3). Control octopus showed a cooking loss of 51.2 ± 5.0% with a variation range between 36.7 and 60.5% in the case of *O. vulgaris*. Cooking losses were significantly lower in *E. cirrhosa* (41.4 ± 8.8%), with values ranging between 24.5 and 59.6%. The typical cooking losses can result from the damaging and solubilization/gelatinization of the connective tissue that connects muscle fibers, followed by disconnection of fibers and dehydration, as reported for squid cooked in hot water [45].

Similarly, Oliveira et al. [46] reported a cooking yield of 52.8% (cooking loss = 47.2%) for *O. vulgaris* water boiled under industrial conditions. Additionally, cooking losses of ca. 50–60% were reported for squid sous-vide cooked at temperatures up to 85 °C [47]. On the other hand, a narrow range, between 32.2 and 49.7%, was reported for non-processed *O. vulgaris* from different geographical locations on the Portuguese coast [27]. These differences might be related to the fresh/frozen condition state of octopus specimens between studies. Accordingly, a significant increase was reported in cooking losses in different cephalopods species (squid, octopus, and cuttlefish) related to the freezing–thawing process [48].

In general, water-added octopus had a higher cooking loss than the control octopus, and values increased with the increase in immersion time in freshwater (Figure 3). After 16 h of immersion in freshwater, the cooking loss of *O. vulgaris* (61.7 ± 6.6%) was significantly higher than that of *E. cirrhosa* (50.0 ± 4.9%), the loss in water-added octopus being about 10% higher than in the control octopus. For comparison purposes, Mendes et al. [27] reported a lower cooking loss of 46.4 ± 2.2% for fresh *O. vulgaris* processed in similar conditions. No significant differences were found in the cooking loss of *O. vulgaris* immersed for 24 and 32 h (ca. 65–66%), or in the case of *E. cirrhosa* immersed for 24 and 36 h (ca. 67–68%). The highest cooking loss determined was 75.4% in the case of *O. vulgaris* and 73.8% for *E. cirrhosa*.

In a previous study, it was reported that tumbling *O. vulgaris* (during 2–6 h) in NaCl solution increased cooking losses from ca. 50% to 60–65%, while lower cooking losses were observed in other cephalopods (squid and cuttlefish) [49]. In another study about the quality of deep-frozen octopus, only ca. 15% of the brands presented cooking loss values within the natural range for *O. vulgaris*, while 40% of the brands showed a cooking loss of 61–65% [5]. Considering these cooking losses and the results reported above, it seems that longer water processing times (≥16 h) or immersion combined with additives and tumbling may be a common practice in the industry.

Considering the average values for the longest immersion treatments (32–36 h), when consumers acquire 1 kg of water-added octopus, which is equivalent to ca. 790 g of non-processed octopus, they end up with ca. 335 g of octopus after cooking. Although the intention of industrial bodies is to maximize profits and not to cause harm, this still affects consumers, as they purchase water at the price of octopus and end up with a product of very reduced weight after cooking. It is important to mention that the weight gain increases even further the cooking loss of octopus. These results show the impact that fraudulent practices may cause in octopus yields after cooking, and consequently, how they can affect consumers’ expectations.

### 3.4. Data Exploration of TDR Results

#### 3.4.1. TDR Results of *O. vulgaris* and *E. cirrhosa*

**Figure 4:**
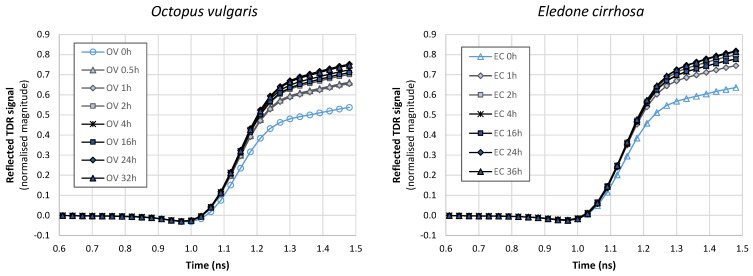
Time domain reflectometry (TDR) data of *Octopus vulgaris* (OV) and *Eledone cirrhosa* (EC) samples immersed in freshwater for different periods of time (0.5 h to 36 h). Data represent the average of all samples from different trials.

TDR data obtained for *O. vulgaris* and *E. cirrhosa* samples were presented in Figure 4. Regarding control samples, TDR curves had a similar profile between the two octopus species, with lower values for *O. vulgaris*, particularly in the region 1.1–1.5 ns. Control samples of *O. vulgaris* had a higher moisture content and a lower electrical conductivity than *E. cirrhosa* samples. However, changes due to moisture content are expected in another region of the plot (between 0.6 and 0.8 ns), while changes due to salt content are mainly expected between 1 and 2.3 ns [50].

In general, an increase in the amount of water in foods increases the dielectric constant and dielectric loss factor of foods, because water (the dipolar molecule) dominantly affects changes in the dielectric properties of food [12]. However, as water is added, other constituents, particularly ionic salts, become diluted and diffuse into the exterior water, thereby decreasing the dielectric loss factor at low frequencies [51]. In a previous piece of research, the increase in water and salt contents of dry-cured hams resulted in a lower reflected signal at the end of the TDR curve [50]. This change in the reflected signal is directly related to the non-linear increase in the loss factor, especially for frequencies below 1 GHz, due to the ionic component of the Hasted–Debye model [50]. In this sense, the differences in TDR results between octopus species might be also related to the different amounts of salts present in muscle tissues.

Octopus (*O. vulgaris* and *E. cirrhosa*) specimens were immersed in freshwater for different periods of time to represent different industrial practices, but also to challenge multivariate methods to detect added-water treatments in octopus samples even for short treatments. The reflected TDR signal increased with the increase in immersion time in freshwater, particularly in the region of 1.1–1.5 ns. It is noticeable that the TDR profile of the control treatment (0 h) is distant from those of water-added treatments, including from short treatments (0.5 h and 1 h). Moreover, some water-added treatments showed overlapping profiles. The trend was similar between species, although with higher TDR signal values in the case of *E. cirrhosa* (Figure 4). The differences observed seem to reflect the decrease in salt content with the water uptake in octopus. The increase in the reflected TDR signal was in line with the decrease in electrical conductivity as the immersion time increased. Previous research studies also reported changes in dielectric properties related to different moisture contents in several food items [12,50,51,52,53].

On the other hand, a study with salmon stored in refrigeration conditions showed that both the dielectric constant and loss factor increased along with storage time [54]. The dielectric loss showed a significant correlation with quality indexes including total volatile basic nitrogen (TVB-N), which is an indicator of the degree of protein degradation, at five selected frequencies (27.12, 40.68, 100, 300, and 915 MHz) [54]. Dielectric properties might be affected by an increase in metabolites and free ions, but also by the loss of free water [54]. A similar effect might have occurred with octopus with the increase in immersion time.

In particular for cuttlefish, TVB-N increased from ca. 7 mg N/100 g (fresh cuttlefish) to 13.85 mg N/100 g after freezing and thawing in refrigeration (ca. 14.5 h) [55]. Thus, considering that octopus has high levels of proteolytic activity [56], it is reasonable to assume that a comparable protein degradation occurs along with immersion time in freshwater (up to 32–36 h/2–3 °C), which can result in the release of charged free amino acids and decrease the water holding capacity of octopus muscle, thus contributing to changes in dielectric properties.

In terms of muscle integrity, the initial condition of fresh and thawed octopus samples submitted to water immersion treatments may differently affect the behavior of water absorption and consequently the dielectric properties. In cuttlefish, after freezing and thawing in refrigeration, a decrease in water holding capacity, an increase in drip loss and in relative free water content, and larger muscle fiber gaps were observed [55]. In octopus, the overall structural organization, the muscle bundle gaping, and the presence of optically empty spaces were considered discrimination parameters of fresh and thawed mantle and arms [57].

The differences in the TDR profiles between control and water-added octopus indicate that the TDR equipment can be calibrated to allow the detection of octopus processed with water. Calibrations can be carried out specifically for each species, as reported previously for *O. vulgaris* [26]. However, it would be advantageous to have a non-destructive method able to detect water addition in octopus, even if the octopus species is not known. This would allow the analysis of octopus by non-trained personnel, and would make the analysis faster in the case of mixed samples.

#### 3.4.2. Multivariate Analysis of TDR Results

**Figure 5:**
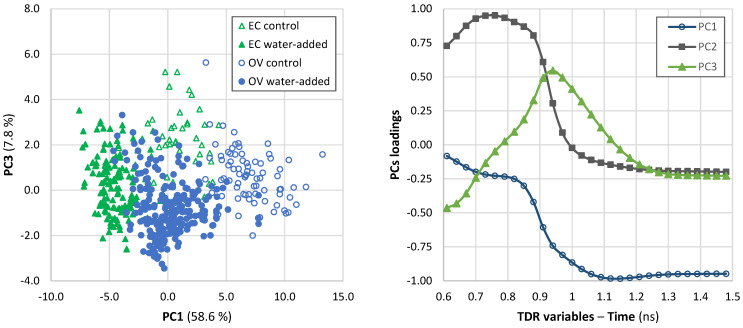
Plot of principal components analysis of TDR data of *Octopus vulgaris* (OV) and *Eledone cirrhosa* (EC) samples and loadings of PC1, PC2, and PC3. Immersion time of water-added treatments varied from 0.5 to 36 h. TDR data between 0.6 and 1.5 ns were included in the multivariate analysis. PC1, PC2, and PC3 explained, respectively, 58.6%, 28.8%, and 7.8% of the variation of original TDR variables.

Considering the complexity of TDR data obtained, data in the range 0.6–1.5 ns were subjected to principal component analysis (PCA) in order to identify groups of samples related to the water-added treatments (Figure 5). The plot with the scores of the first and third principal components (PC1 and PC3) shows that *O. vulgaris* samples from water-added group are partially overlaid with *E. cirrhosa* samples (control and water-added). For each species, control samples were somehow separated from water-added ones. This separation is mainly due to PC1 in the case of *O. vulgaris*, while for *E. cirrhosa*, it is based on both PC1 and PC3. Different PC1 scores are explained by variations in TDR data between 1.1–1.5 ns, while variations around 0.9–1.0 ns are reflected in PC3 scores (Figure 5). Variations in TDR data in the region 0.6–0.9 ns did not seem to have any relevance for the distinction of groups. Those variations were reflected in PC2, but it did not show useful clustering, and for that reason this PC was not represented in the plot. Still, the PCA analysis did not allow an adequate separation of the groups of interest.

### 3.5. Calibration and Validation of TDR Analysis

**Figure 6:**
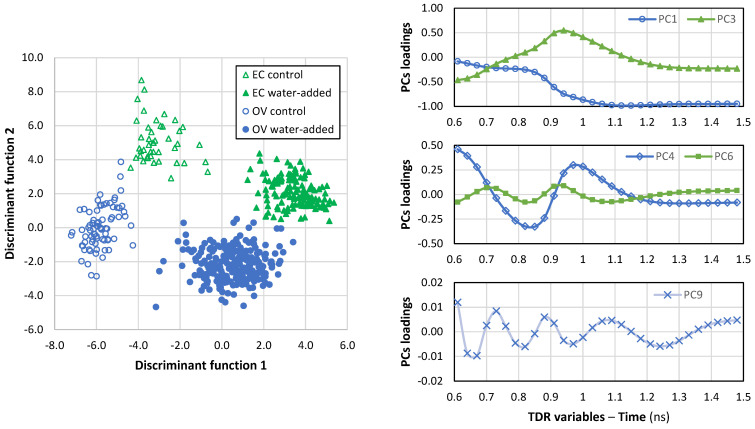
Plot of the discriminant analysis model of TDR data of *Octopus vulgaris* (OV) and *Eledone cirrhosa* (EC) samples and loadings of PCs with the highest factor structure coefficients. The immersion time of water-added treatments varied between 0.5 and 36 h. The discriminant analysis was performed using the principal components obtained from the TDR data, and the plot shows the scores of discriminant functions 1 and 2. The explained variance accounted for was 61.4 and 37.3% by discriminant functions 1 and 2, respectively.

Taking into account the data dispersion and samples overlapping in the PCA, a linear discriminant analysis was then performed on all principal components obtained from TDR data between 0.6 and 1.5 ns. In a first approach, all samples were used for training the model (model 1). Discriminant analysis clearly showed four groups of samples separated in the plot (Figure 6). The loadings of the most relevant PCs were presented in Figure 6. The first discriminant function mostly discriminated between control and water-added samples, and PC1 had the highest (in absolute value) factor structure coefficient (PC1: −0.60), i.e., the highest correlation between the variables (PCs) and the discriminant function. Hence, variations in TDR data between 1.1–1.5 ns were on the basis of the discrimination between control and water-added treatments. The second discriminant function provided discrimination between the two octopus species. In particular, the factor structure coefficients were higher (in absolute value) for PC1, PC3, PC4, PC6, and PC9 (the factor structure coefficients were −0.23, 0.27, 0.18, 0.19, and 0.10, respectively).

A classification model based in the TDR data of all samples was developed to predict groups membership, and the model showed that 98.8% of samples were correctly classified (Table 2). Misclassification was low; nevertheless, it needs to be taken into consideration that in model 1, all samples were used for training.

In a second approach, some of the samples were used to create the model (model 2), and the remaining samples were used to challenge model 2 to classify new samples. In this model, only four trials of *O. vulgaris* and two trials of *E. cirrhosa* were used for the calibration, and about one third of the samples (two trials of *O. vulgaris* and one trial of *E. cirrhosa*) were selected for the validation. The results showed that in model 2, all samples used for training and 87.5% of samples used for testing were correctly classified (Table 2). In particular, for samples used for testing, control samples were never classified as water-added samples. Additionally, all *E. cirrhosa* samples were correctly classified. The number of samples that were misclassified in each group was reduced. Only five samples of *O. vulgaris* water-added samples were misclassified as *O. vulgaris* control samples, and a total of 14 samples were misclassified in terms of species.

Regarding cross-validation, results showed that 98.6% of samples were correctly classified (Table 2). The number of samples that were misclassified in each cross-validated group (5 groups) was reduced (≤3%). In this sense, the results indicate that the data represent well the actual population, and thus the model can be generalized.

Although some misclassification was observed (mainly in terms of species), the results evidenced the potential of TDR to detect water addition in octopus. Moreover, the results obtained with the classification model indicate that TDR can be used for detection of water addition in *O. vulgaris* and *E. cirrhosa*, including if it is not possible for the operator to distinguish between these two species (e.g., mixed species). The use of rapid and non-destructive methods allows a prompt evaluation of a higher number of samples without compromising the integrity of octopus products, contributing to the sustainable use of resources. It can also contribute to improving the assessment of octopus quality by the industry, and to verifying compliance with legislation, thus encouraging fair trade practices.

## 4. Conclusions

Baseline levels of moisture content, M/P ratio, cooking loss, and electrical conductivity were determined for *O. vulgaris* and *E. cirrhosa*. A moisture content limit of 83.0 g/100 g was proposed for *E. cirrhosa* for conformity assessment of commercial products, taking into consideration the highest value of the 95% confidence interval and the 5% limit of added water.

Octopus increased in weight about 5% in short immersion treatments (0.5–1 h), and water addition must be declared on the labels of products above this limit. Thus, industrial bodies should monitor octopus weight during processing to guarantee that the required information is included in products’ labels.

The longest immersion treatments (24–36 h) had similar moisture contents and cooking losses to those reported for several commercial octopus products suspected of abusive water addition. The increase in weight was higher in *O. vulgaris* than in *E. cirrhosa* immersed in freshwater for 32 and 36 h, respectively, although similar cooking losses were observed. The incorporation of water in octopus by industries has no technological function and deceives consumers, because they buy water at the price of octopus and the octopus yields decrease considerably after cooking.

The results showed the potential of the TDR technology combined with multivariate methods to rapidly detect if *O. vulgaris* or *E. cirrhosa* specimens were processed with added water. The classification model developed showed that TDR is able to detect added water in octopus, even in the case of low water uptake (5–10%, immersion during 1–2 h). The analysis is performed within a few seconds, and it does not damage octopus products. This technology, based on changes in dielectric properties, could be used either to prove the authenticity of octopus or to detect fraudulent practices by suppliers/industrial bodies regarding added water.

The use of salt, polyphosphates or other additives applied by the industry is known to interfere with the dielectric properties of seafood [16,31,52]. In this sense, in future work, it is necessary to assess how the models are affected by additives, and whether it is necessary to adjust the models for an accurate classification of octopus products. It is also important to prove the adequacy of the models with commercial samples.

## Figures and Tables

**Table 1:** Moisture and protein contents in several octopus species from different locations (data from the literature).

Octopus Species	Moisture (g/100 g)	Protein (g/100 g)	M/P Ratio	Origin and Other Details	Reference
*Octopus vulgaris*	80.7 ± 1.6 (76.2–85.4)	16.6 ± 1.5 (12.0–19.1)	3.9–6.9 4.9 ± 0.6	Portuguese coast; fresh non-processed samples	Mendes et al. [27]
*Octopus vulgaris*	Viana do Castelo: 78.2–81.4 Cascais: 78.0–80.2 Tavira: 76.5–80.5	Viana do Castelo: 16.1–18.4 Cascais: 17.0–18.3 Tavira: 17.1–19.8		Portuguese coast; *n* = 195	Rosa et al. [33]
*Octopus vulgaris*		12.7–17.5		Cascais, Portugal	Leonardo [38]
*Octopus vulgaris*	81.10 ± 0.68	12.80 ± 0.38	6.3 *	Aydın, Turkey; *n* = 6	Özalp and Karakaya [32]
*Octopus vulgaris*	S: 83.41 ± 0.08 A: 82.53 ± 0.13 W: 80.71 ± 1.18	S: 14.83 ± 0.67 A: 14.78 ± 1.0 W: 15.28 ± 0.21	S: 5.6 * A: 5.6 * W: 5.3 *	Eastern Mediterranean sea; *n* ≥ 3	Ozogul et al. [39]
*Octopus vulgaris*	NEA: 88.7 ± 3.6 (84.5–92.4) NWA: 81.5 ± 1.0 (80.7–82.8) ECA: 91.7 ± 4.1 (88.4–97.4) WCA: 85.9 ± 0.6 (85.6–86.7) PO: 89.2 ± 0.6 (88.5–89.9) MS: 91.0 ± 2.8 (88.5–94.3)			Markets from the NW region of Portugal; NEA, NWA, ECA, and WCA, PO: *n* = 8 MS: *n* = 5	Torrinha et al. [40]
*Octopus vulgaris*	NEA: 87.5 (82.8–92.4) NWA: 81.5 (78.0–83.9) ECA: 90.2 (83.8–97.4) WCA: 87.4 (85.6–90.7) PO: 89.8 (88.5–93.1) MS: 90.6 (88.5–94.3)			Markets from the NW region of Portugal; *n* = 144	Oliveira et al. [41]
*Octopus vulgaris*	80.4 ± 1.5	15.8 ± 3.4	5.1 *	Fish market in Thessaloniki, Greece	Zlatanos et al. [42]
*Octopus vulgaris*	83.6 ± 2.2	77.8 ± 3.2 DW		Wild octopus captured in Canary Islands, Spain	Estefanell et al. [34]
*Octopus vulgaris*	WC: 85.2 ± 0.6 BC: 85.9 ± 0.4 DB: 84.7 ± 1.0 WC + DB: 84.1 ± 0.6 BC + DB: 84.0 ± 0.9	WC: 81.9 ± 1.5 DW BC: 78.3 ± 0.9 DW DB: 84.0 ± 0.6 DW WC + DB: 82.7 ± 2.1 DW BC + DB: 83.9 ± 0.4 DW		Captured in Canary Islands, Spain; fed with different diets for 8 weeks (*n* = 8 for each diet)	Estefanell et al. [34]
*Octopus vulgaris*	81.87 ± 2.12	14.230 ± 0.225	5.8 *	Wild octopus; Ionian Sea, Southern Italy	Prato et al. [35]
*Octopus vulgaris*	DG I: 82.05 ± 1.28 DG II: 81.73 ± 2.87 DG III: 81.42 ± 2.41 DG IV: 81.58 ± 1.82 DG V: 81.73 ± 2.51	DG I: 14.376 ± 0.221 DG II: 14.718 ± 0.256 DG III: 14.884 ± 0.147 DG IV: 14.740 ± 0.292 DG V: 14.690 ± 0.281	DG I: 5.7 * DG II: 5.6 * DG III: 5.5 * DG IV: 5.5 * DG V: 5.6 *	Captured in Ionian Sea, Southern Italy; Cultured octopus, with different diets (*n* = 10 for each diet)	Prato et al. [35]
*Octopus vulgaris*, *Octopus mimus*, and *Octopus* *cyanea*	83.4–90.1	6.5–14.8	9.5 ± 1.9 (5.6–13.8)	Frozen octopus available in markets from Portugal; *n* = 25 (23 samples of *Octopus vulgaris*)	Mendes et al. [5]
*Octopus maya*	ECA: 92.2 ± 2.0 (90.5–94.9) WCA: 86.1 ± 1.7 (84.0–88.0)			Markets from the NW region of Portugal; ECA and WCA: *n* = 8	Torrinha et al. [40]
*Octopus maya*	WCA: 89.1 (84.0–94.9)			Markets from the NW region of Portugal; *n* = 48	Oliveira et al. [41]
*Eledone cirrhosa*	76	16.2	4.7 *	Bay of Biscay; *n* = 3	Spitz et al. [36]
*Eledone cirrhosa*	immature: 81.19 in mantle 80.21 in arms mature: 80.04 in mantle 78.54 in arms	immature: 15.67 in mantle 16.64 in arms mature: 15.85 in mantle 17.89 in arms	5.2 * 4.8 * 5.0 * 4.4 *	Galician shelf; summer; *n* ≥ 30	Ruiz-Capillas et al. [37]
*Eledone cirrhosa*	NEA: 84.1 ± 1.6 (82.0–85.9)			Markets from the NW region of Portugal; NEA: *n* = 8	Torrinha et al. [40]
*Eledone cirrhosa*	NEA: 83.1 (79.6–85.8)			Markets from the NW region of Portugal; *n* = 24	Oliveira et al. [41]
*Eledone moschata*	S: 84.64 ± 0.39 A: 83.12 ± 0.21 W: 82.79 ± 0.20	S: 12.21 ± 0.62 A: 14.32 ± 0.36 W: 14.50 ± 0.42	S: 6.9 * A: 5.8 * W: 5.7 *	Eastern Mediterranean sea; *n* ≥ 3	Ozogul et al. [39]

* values calculated based on average values of moisture and protein contents. Abbreviations: summer (S); autumn (A); winter (W); Northeast Atlantic Ocean (NEA); Northwest Atlantic Ocean (NWA); Eastern Central Atlantic Ocean (ECA); Western Central Atlantic Ocean (WCA); Pacific Ocean (PO); Mediterranean Sea (MS); diet-white crab (WC); diet-blue crab (BC); diet-discarded bogue (DB); diet-white crab and discarded bogue (WC + DB); diet-blue crab and discarded bogue (BC + DB); fed on crab, bogue and mussels (DG I); fed on bogue (DG II); fed on mussels (DG III); fed on crab (DG IV); fed on crab and two-banded sea bream; dry weight (DW).

**Table 2:** Classification results (number of samples and percentage) of discriminant analysis of TDR data of *Octopus vulgaris* and *Eledone cirrhosa* samples. The immersion time of water-added treatments varied between 0.5 and 36 h. The discriminant analysis was performed in the principal components obtained from the TDR data (between 0.6 and 1.5 ns). The same a priori classification probability was chosen for all groups. Correct classifications are shown in bold. In model 1, 98.8% of samples (used for training) were correctly classified. In model 2, all samples used for training and 87.5% of samples used for testing were correctly classified. In the cross validation, 98.6% of samples were correctly classified.

	Actual Groups	Predicted Groups Membership				Total
		*O. vulgaris* Control	*O. vulgaris* Water-Added	*E. cirrhosa* Control	*E. cirrhosa* Water-Added	
Model 1—all trials/samples were used for training						
Samples used for training	*O. vulgaris* control	**74** **(97.4%)**	0 (0.0%)	2 (2.6%)	0 (0.0%)	76
	*O. vulgaris* water-added	1 (0.4%)	**227** **(98.3%)**	0 (0.0%)	3 (1.3%)	231
	*E. cirrhosa* control	0 (0.0%)	0 (0.0%)	**44** **(100.0%)**	0 (0.0%)	44
	*E. cirrhosa* water-added	0 (0.0%)	0 (0.0%)	0 (0.0%)	**132** **(100.0%)**	132
	Total	75	227	46	135	
Model 2—1/3 of the trials were used for testing (4 trials of *O. vulgaris* and 2 trials of *E. cirrhosa* were used for training)						
Samples used for training	*O. vulgaris* control	**50** **(100.0%)**	0 (0.0%)	0 (0.0%)	0 (0.0%)	50
	*O. vulgaris* water-added	0 (0.0%)	**153** **(100.0%)**	0 (0.0%)	0 (0.0%)	153
	*E. cirrhosa* control	0 (0.0%)	0 (0.0%)	**32** **(100.0%)**	0 (0.0%)	32
	*E. cirrhosa* water-added	0 (0.0%)	0 (0.0%)	0 (0.0%)	**96** **(100.0%)**	96
	Total	49	153	32	96	
Samples used for testing	*O. vulgaris* control	**25** **(96.2%)**	0 (0.0%)	1 (3.8%)	0 (0.0%)	26
	*O. vulgaris* water-added	5 (6.4%)	**60** **(76.9%)**	0 (0.0%)	13 (16.7%)	78
	*E. cirrhosa* control	0 (0.0%)	0 (0.0%)	**12** **(100.0%)**	0 (0.0%)	12
	*E. cirrhosa* water-added	0 (0.0%)	0 (0.0%)	0 (0.0%)	**36** **(100.0%)**	36
	Total	30	60	13	49	
Cross validation (5-fold)						
Samples used for testing	*O. vulgaris* control	**74** **(97.4%)**	0 (0.0%)	2 (2.6%)	0 (0.0%)	76
	*O. vulgaris* water-added	1 (0.4%)	**226** **(97.8%)**	0 (0.0%)	4 (1.7%)	231
	*E. cirrhosa* control	0 (0.0%)	0 (0.0%)	**44** **(100.0%)**	0 (0.0%)	44
	*E. cirrhosa* water-added	0 (0.0%)	0 (0.0%)	0 (0.0%)	**132** **(100.0%)**	132
	Total	75	226	46	136	

## Data Availability

The datasets generated for this study are available on request to the corresponding author.
